# The impact of mental vulnerability on the relationship between cardiovascular disease and depression

**DOI:** 10.1192/j.eurpsy.2020.20

**Published:** 2020-02-14

**Authors:** Terese Sara Høj Jørgensen, Marie Kim Wium-Andersen, Martin Balslev Jørgensen, Merete Osler

**Affiliations:** 1 Center for Clinical Research and Prevention, Bispebjerg and Frederiksberg Hospital, University of Copenhagen, Copenhagen, Denmark; 2 Department of Public Health, Section of Social Medicine, University of Copenhagen, Copenhagen, Denmark; 3 Department O, Psychiatric Center Copenhagen, and Institute of Clinical Medicine, University of Copenhagen, Copenhagen, Denmark; 4 Department of Public Health, Section of Epidemiology, University of Copenhagen, Copenhagen, Denmark

**Keywords:** cardiovascular disease, depression, epidemiology, interaction analyses, mental vulnerability

## Abstract

**Background.:**

The mechanisms linking cardiovascular disease (CVD) and depression are still not established. We investigated the impact of mental vulnerability on the relationship between CVD and depression.

**Methods.:**

A total of 19,856 individuals from five cohorts of random samples of the background population in Copenhagen were followed from baseline (1983–2011) until 2017 in Danish registries. Additive hazard and Cox proportional hazard models were used to analyze the effects of confounding by mental vulnerability as well as interactions between mental vulnerability and CVD on the risk of depression.

**Results.:**

During follow-up, 15.3% developed CVD, while 18.1% experienced depression. A strong positive association between CVD and depression (hazard ratio: 3.60 [95% confidence intervals (CI): 3.30; 3.92]) corresponding to 35.4 (95% CI: 31.7; 39.1) additional cases per 1,000 person-years was only slightly attenuated after adjustment for mental vulnerability in addition to other confounders. Synergistic interaction between CVD and mental vulnerability was identified in the additive hazard model. Due to interaction between CVD and mental vulnerability, CVD was associated with 50.9 more cases of depression per 1,000 person-years among individuals with high mental vulnerability compared with individuals with low mental vulnerability.

**Conclusions.:**

Mental vulnerability did not explain the strong relationship between CVD and depression. CVD was associated with additional cases of depression among individuals with higher mental vulnerability indicating that this group holds the greatest potential for intervention, for example, in rehabilitation settings.

## Introduction

Today, a large proportion of patients survive cardiovascular disease (CVD) due to considerable improvements in treatment; however, CVD patients are at high risk of developing mental disorders, such as anxiety and depression [1–5]. Health behavioral patterns and biological pathways including exogenous stressors and physiological stress response have been suggested to partly explain the relationship between CVD and depression [2,6]. Yet, the linking mechanisms are still not established, and few studies have examined how psychosocial factors, including personality traits and negative emotions, may contribute to the risk of developing depression after CVD.

Mental vulnerability may explain the link between CVD and depression. Mental vulnerability—a tendency toward experiencing interpersonal problems and psychosomatic and mental symptoms—can be measured on different scales. The Danish mental vulnerability scale is a validated measure of long-term mental vulnerability and comparable with other international measures [7,8]. The original scale was developed in the 1960s to assess mental fitness for Danish military service, but the current version has been shortened to include 12 items. The Danish mental vulnerability scale measures a symptom state and a personality trait component [7] and the trait component may explain why mental vulnerability is a risk factor for developing a number of adverse health conditions including depression and ischemic heart disease [7,9–16]. Accordingly, scores on the mental vulnerability scale have been found to correlate with the trait of neuroticism and type D personality [9,10]. Thus, mental vulnerability could be an important confounder of the association between CVD and depression. It is, however, also likely that depression following CVD mainly occurs in patients who are mentally vulnerable because of greater susceptibility to stressful situations such as experiencing CVD. This latter explanation of mental vulnerability interacting with CVD on the risk of depression would be illustrated by differences in the associations between CVD and depression between individuals with low and high levels of mental vulnerability. So far, mental vulnerability has neither been investigated as a confounder nor as a modifier (interaction) of the relationship between CVD and depression.

In this study, we investigated whether mental vulnerability explained (confounded) or interacted with the effect of CVD for the risk of developing depression.

## Material and Methods

### Data source

**Figure A1.**
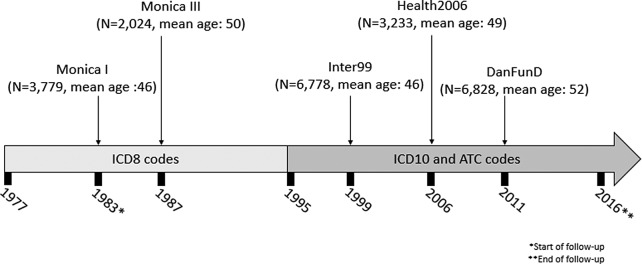
Timeline for International Classification of Disease version 8 and 10 (ICD8 and ICD10) codes and Anatomic Therapeutical Chemical (ATC) codes to obtain information on cardiovascular disease and depression for the specific sub-cohorts’ baseline.

We used pooled data from five cohorts from the Center for Clinical Research and Prevention; Monica I and III, Inter99, The Regional Health Survey 2006/2007 (Health2006), and the Danish Study of Functional Disorders (DanFunD) part 2 collected between 1983 and 2011. All the cohorts were based on random samples of the background population in the Copenhagen region, Denmark. The data collection included questionnaire data and physical examinations. The aims of the different cohorts were to monitor trends in CVD in different countries in Monica I & III, to evaluate the effect of lifestyle intervention on cardiovascular risk in Inter99, to study lifestyle-related chronic conditions in Health 2006, and to study functional disorders in the general population in DanFunD. Even though coronary heart disease and functional somatic disorder were the initial analytical focus of the data collections, many other health conditions have been investigated using data from these cohorts. All study participants provided written informed consent and the study protocols for data collections were approved by the Danish Data Protection Agency. Baseline participation rates ranged from 29 to 79% (see Figure A1 for further details) [17,18]. We linked the survey data with the following six national registers: the Danish Civil Registration System, the Danish Income Register, the Population Education Register, the Danish National Patient Register, the Psychiatric Central Register, and the Danish National Prescription Register to obtain register-based information [19–24].

**Figure A2.**
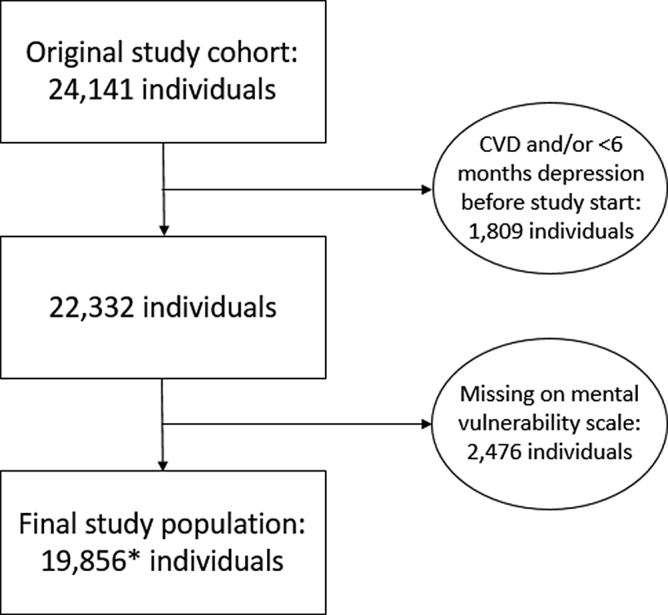
Flow chart of selection of study population. Footnote: 620 individuals included into the study again at the time of a cardiovascular disease (CVD) after depression.

Figure A2 shows the selection of the final study population covering 19,856 Danish men and women. Strict selection criteria of no CVD prior to baseline and no depression six months prior to baseline were applied to ensure temporality between the exposure and the outcome.

### Main variables

CVD was the main exposure. CVD was measured by in- and outpatient hospital diagnoses in the Danish National Patient Registry based on the 8th and 10th revision of the International Classification of Diseases (ICD8 and ICD10) codes covering ischemic heart disease (ICD8: 410–414 and ICD10: I20–25) and stroke (ICD8: 430–438 and ICD10: I60–69, G45).

Depression was the outcome. Depression was measured by in- and outpatient contacts with ICD8 and ICD10 codes (ICD8: 296.09, 296.29, 298.09 or 300.49, and ICD10: F32–33) in the Danish National Patient Registry and/or the Psychiatric Central Register and/or purchase of antidepressants registered by Anatomic Therapeutical Chemical (ATC) codes (N06A) in the Danish National Prescription Registry.

Figure A1 presents a timeline for ICD8, ICD10, and ATC codes used to obtain information on CVD and depression for each of the specific sub-cohorts.

Mental vulnerability was investigated as a potential confounding factor of the relationship between CVD and depression and/or whether it interacted with CVD for development of depression. Mental vulnerability was assessed by the Danish mental vulnerability scale obtained at baseline through questionnaire data from the surveys [17]. We applied the 12-item mental vulnerability subscale, which has a high correlation of 0.93 with the original 22-item mental vulnerability scale (see items in Table 1). The 12-item mental vulnerability scale was divided into three categories by low (0–2 points), moderate (3–4 points), and high (5–12 points). This categorization is based on previous studies of ischemic heart disease and depression [9,10].

**Table 1.** The 22-item questionnaire for the test of mental vulnerability [7] with the questions included in the 12-item mental vulnerability subscale (used in this study) highlighted in bold

**1. Do your hands easily shake?**
**2. Do you often suffer from loss of appetite?**
3. Do you often suffer from severe headache?
**4. Do you often suffer from sleeplessness?**
5. Do you often have anxiety attacks?
**6. Do you often feel very tired?**
**7. Do you often take medicine, such as headache tablets, sleeping pills, tranquillizers or the like?**
**8. Do you often have pain in different parts of your body, for example, your stomach, neck, back, or chest?**
9. Do you suffer from bad nerves?
**10. Do you often suffer from fits of dizziness?**
11. Do you believe that noise bothers you more than it does most other people?
12. Are you nearly always in a bad mood?
13. Is it difficult for you to concentrate on your work when someone is watching you?
**14. Does your heart often beat very fast for no particular reason?**
15. Do you often feel unwell?
**16. Is it difficult for you to make friends?**
17. Is it difficult for you to accept that other people decide over you?
18. Do you prefer to keep to yourself?
**19. Do small things get on your nerves?**
**20. Do you constantly have thoughts, which trouble and worry you?**
21. Are you very shy or sensitive?
**22. Do you usually feel misunderstood by other people?**

### Covariates

Age (continuous variable) and gender (female as reference) were included from the Danish Civil Registration System. Educational level was included from the Population Education Register and categorized as basic education (7–9 grade of obligatory schooling), medium education (high school degree/vocational training) as reference, higher education (more than high school degree), and missing. Marital status was included from the Danish Civil Registration System and categorized as married (reference), unmarried, divorced, or widow/widower. Information on alcohol consumption (number of alcoholic drinks per day), smoking status categorized by never (reference), former or current smoker, and physical activity categorized by sedentary, light (reference), moderate, and hard exercise were obtained from baseline survey questionnaires. Body mass index (BMI) was identified from physical examination of height and weight at baseline and categorized as <21.5 equal to underweight, 21.5–25.0 equal to normal weight (reference), and >25 equal to overweight. Systolic blood pressure was based on physical examination and categorized in quintiles (first quintile as reference). Missing values for alcohol use, smoking status, physical activity, and BMI were imputed using multiple imputations based on age, gender, and marital status, which built on the assumption that these variables were missing completely at random. Type 1 and Type 2 diabetes mellitus were identified up to baseline by ICD8 and ICD10 codes (250; E10-E14) from the Danish National Patient register. Previous depression up to six months before baseline was defined by the previous described ICD8, ICD10, and ATC codes.

### Statistical methods

Descriptive statistics of baseline characteristics for CVD and development of depression were conducted using means and standard deviation for continuous variables and frequencies with percentages for categorical variables.

We analyzed the associations between CVD and development of depression using an additive hazard model to estimate risk differences (additional cases) with 95% confidence intervals (CIs) [25], and the more commonly used multiplicative Cox proportional hazard (PH) model to estimate hazard ratios (HRs) with 95% CIs. Time from baseline was the underlying time scale in both models and CVD was included as a time-dependent variable. Individuals were followed from baseline and until development of depression, emigration, death, or end of follow-up (July 11, 2017). Model assumptions for the additive hazard model (time-constant hazard differences) and the Cox PH model (time-constant hazards ratios) were fulfilled.

The association between CVD and depression was analyzed in three models with different levels of adjustments; Model 1: unadjusted, Model 2: adjusted for potential confounders (age, sex, educational level, marital status, smoking status, alcohol consumption, physical activity, BMI, systolic blood pressure, diabetes mellitus, previous depression, and study cohorts), and Model 3: Model 2 with additional adjustment for the mental vulnerability scale. We calculated whether mental vulnerability explained the association between CVD and depression by comparing the adjusted regression models with and without adjustment for the mental vulnerability scale. We assessed the impact of adjustment for mental vulnerability both in the additive hazard model (A model) and in the Cox PH model (M model). The proportion of excess risk accounted for by including mental vulnerability (Model 3) in the covariate adjusted model (Model 2) was calculated using the formula (Amodel2 – Amodel3/Amodel2) for the additive model and the formula (Mmodel2 – Mmodel3)/(Mmodel2 – 1) for the multiplicative model [26].

Interaction between exposures for an outcome can be defined by departure from multiplicativity or additivity. Psychiatric and epidemiological literature encourage test of both additive and multiplicative interaction models [25,27–29]. However, interaction on the additive scale is often thought of as more relevant to public health and clinical decision making because the additive scale shows absolute number of patients. In contrast, the multiplicative scale shows the relative risks, which may only translate into few individuals in absolute numbers. Thus, the results from the additive hazard analysis will be valued above the results from the Cox PH analysis in this study, but the results from the Cox PH model will be provided as this model is more widely used. To test whether the association between CVD and depression differed between individuals with low, moderate, and high mental vulnerability, global tests of interaction between CVD and mental vulnerability for development of depression were conducted. To illustrate the potential interaction between CVD and mental vulnerability, a joint exposure variable combining CVD and levels of mental vulnerability was analyzed with no CVD and low mental vulnerability as the reference group.

Supplementary analyses were performed for (a) depression defined by prescription medicine or hospitalization, respectively and (b) individuals without prior depression.

Statistical analyses were conducted in the statistical software packages Stata and R.

## Results

In this study, 19,856 Danish men and women were followed for a total of 254,250.5 person-years. Baseline characteristics of individuals by mental vulnerability are shown in Table A1. Among the study population, 77% scored low (0-2), 15% scored moderate (3-4), while 8% had a high score (5+). Table 2 provides distribution of covariates and mental vulnerability for the study participants with and without CVD (main exposure) and depression (outcome), respectively. During follow-up, 15.3% were diagnosed with CVD and 18.1% were diagnosed with depression (Table 2).

**Table 2.** Incident cardiovascular disease and depression during follow-up based on baseline characteristics for study population (N=19,856)

	Incident cardiovascular disease^a^		Depression during follow-up^a^	
	No	Yes	No	Yes
**All**	16,809 [84.7]	3,047 [15.3]	16,260 [81.9]	3,596 [18.1]
**Age**				
Mean	47.2 [12.0]	50.7 [10.5]	48.0 [12.0]	46.9 [11.0]
**Gender**				
Females	9,029 [87.3]	1,315 [12.7]	8,125 [78.6]	2,219 [22.4]
Males	7,780 [81.8]	1,732 [18.2]	8,135 [85.5]	1,377 [14.5]
**Education**				
Short	8,966 [85.2]	1,561 [14.8]	8,761 [83.2]	1,766 [16.8]
Medium	3,648 [78.4]	1,003 [21.6]	3,477 [74.8]	1,174 [25.2]
Long	4,024 [90.3]	431 [9.7]	3,861 [86.7]	594 [13.3]
Missing	171 [76.7]	52 [23.3]	161 [72.2]	62 [27.8]
**Marital status**				
Married	11,410 [83.5]	2,246 [16.5]	11,220 [82.2]	2,436 [17.8]
Unmarried	3,160 [92.0]	276 [8.0]	2,884 [83.9]	552 [16.1]
Divorced	1,856 [82.3]	399 [17.7]	1,768 [78.4]	487 [21.6]
Widow/widower	383 [75.3]	126 [24.7]	388 [76.2]	121 [23.8]
**Cohorts**				
Monica I	2,449 [66.3]	1,244 [33.7]	2,577 [69.8]	1,116 [30.2]
Monica III	1,197 [70.9]	491 [29.1]	1,119 [66.3]	569 [33.7]
Inter99	5,032 [84.5]	923 [15.5]	4,571 [76.8]	1384 [23.2]
Health2006	2,482 [90.6]	259 [98.5]	2,383 [86.9]	358 [13.1]
DanFunD	5,649 [97.8]	130 [2.2]	5,610 [97.1]	169 [2.9]
**Smoking**				
Never	6,802 [89.2]	827 [10.8]	6,667 [87.4]	962 [12.6]
Former	4,770 [86.2]	765 [13.8]	4,743 [85.7]	792 [14.3]
Current	5,237 [78.3]	1,455 [21.7]	4,850 [72.5]	1,842 [27.5]
**Alcohol consumption**				
Mean [SD]	1.2 [1.5]	1.4 [1.8]	2.9 [1.4]	2.8 [1.4]
**Physical activity**				
Sedentary	9,571 [84.6]	1,747 [15.4]	9,272 [81.9]	2,046 [18.1]
Light	3,126 [80.6]	755 [19.4]	2,904 [74.8]	977 [25.2]
Moderate	3,869 [88.3]	511 [11.7]	3,832 [87.5]	548 [12.5]
Hard exercise	243 [87.7]	34 [12.3]	252 [91.0]	25 [9.0]
**Body mass index**				
Underweight [< 21.5]	6,678 [85.5]	2,225 [14.5]	6,386 [81.8]	1,426 [18.3]
Normal weight [21.5–25.0]	2,718 [88.6]	495 [11.4]	2,418 [78.8]	650 [21.2]
Overweight [> 25]	7,413 [82.6]	327 [17.4]	7,456 [83.1]	1,520 [16.9]
**Systolic blood pressure**				
1^st^ quintile	3,679 [89.5]	434 [10.5]	3,259 [79.2]	854 [20.8]
2^nd^ quintile	3,782 [87.4]	545 [12.6]	3,530 [81.6]	797 [18.4]
3^rd^ quintile	3,577 [84.5]	658 [15.5]	3,463 [81.8]	772 [18.2]
4^th^ quintile	3,002 [82.8]	624 [17.2]	3,028 [83.5]	598 [16.5]
5^th^ quintile	2,769 [77.9]	786 [22.1]	2,980 [83.8]	575 [16.2]
**Diabetes**				
No	16,625 [84.8]	2,985 [15.2]	16,065 [81.9]	3,545 [18.1]
Yes	184 [74.8]	62 [25.2]	195 [79.3]	51 [20.7]
**Previous depression**				
No	15,594 [84.2]	2,924 [15.8]	15,303 [82.6]	3,215 [17.4]
Yes	1,215 [90.8]	123 [9.2]	957 [71.5]	381 [28.5]
**12-item Mental Vulnerability Scale**				
Low [0-2]	13,013 [85.4]	2,225 [14.6]	13,090 [85.9]	2,148 [14.1]
Moderate [3-4]	2,471 [83.3]	495 [16.7]	2,175 [73.3]	791 [26.7]
High [5+]	1,325 [80.2]	327 [19.8]	995 [60.2]	657 [39.8]

* The 620 individuals who reenter the study after recovery of depression before cardiovascular disease are not included in this table.

**Figure 1.**
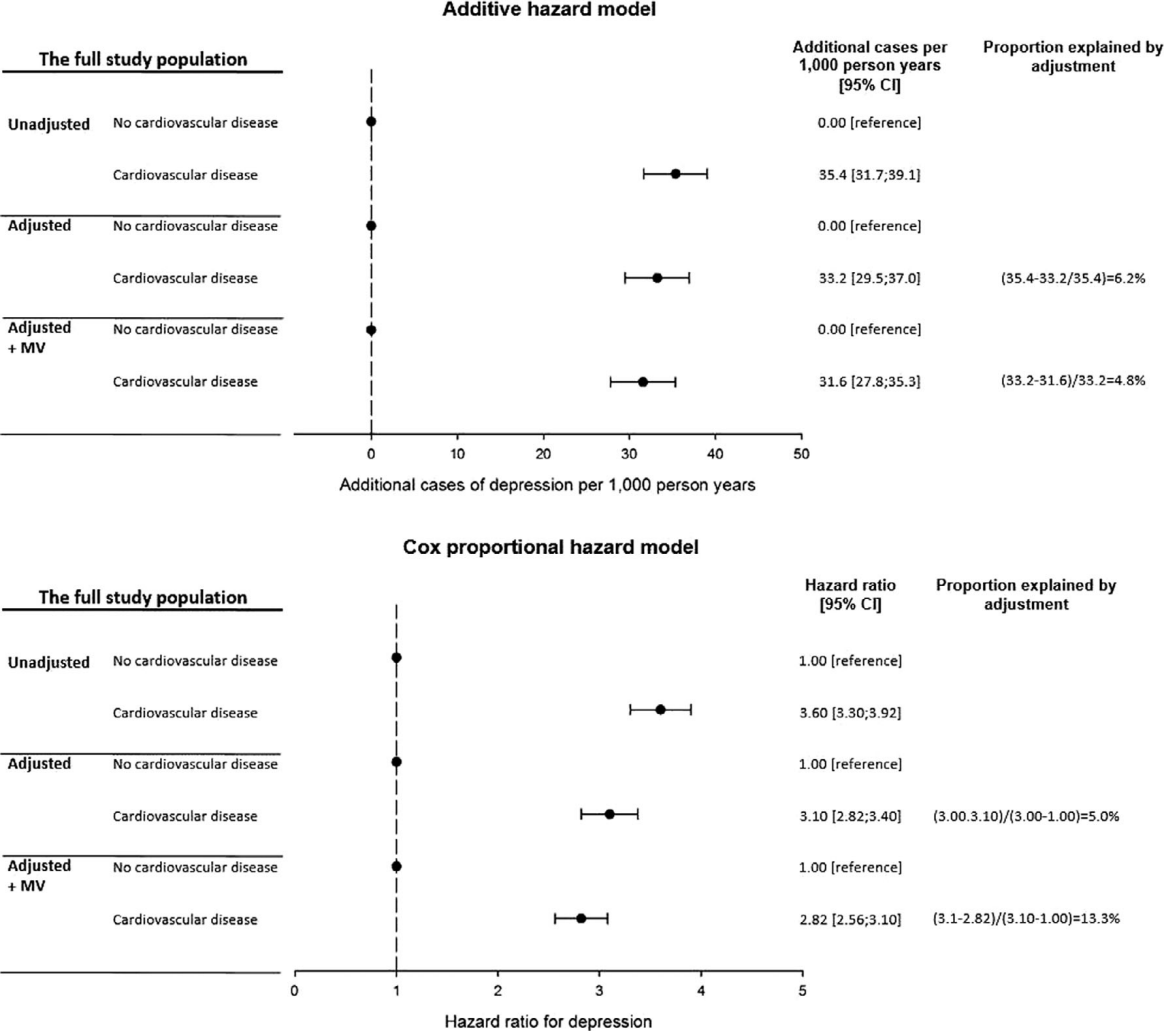
Associations between onset of cardiovascular disease and depression during follow-up by three levels of adjustments: (1) unadjusted, (2) adjusted for (age, sex, educational level, marital status, smoking status, alcohol consumption, physical activity, body mass index, systolic blood pressure, diabetes, prior depression, and cohorts), and (3) additional adjustment for mental vulnerability (MV).

Individuals who were diagnosed with CVD were associated with 3.60 (95% CI: 3.30; 3.92) times higher risk of depression, which corresponded to 35.4 (95% CI: 31.7; 39.1) additional cases of depression per 1,000 person-years in the unadjusted analyses (Figure 1). After adjustment for age, sex, educational level, marital status, smoking, alcohol consumption, physical activity, BMI, systolic blood pressure, diabetes, prior depression, and study cohort, the risk estimate was attenuated with 6.2% in the additive model to 33.2 (95% CI: 29.5; 37.0) additional cases per 1,000 person-years (corresponding to a HR of 3.10 [95% CI: 2.82; 3.40]). Further adjustment for mental vulnerability explained 4.8% of the confounder adjusted association between CVD and depression in the additive hazard model. The fully adjusted risk estimate was 31.6 (95% CI: 27.8; 35.3) additional cases of depression per 1,000 person-years (corresponding to a HR of 2.82 [95% CI: 2.56; 3.10]).

**Figure 2.**
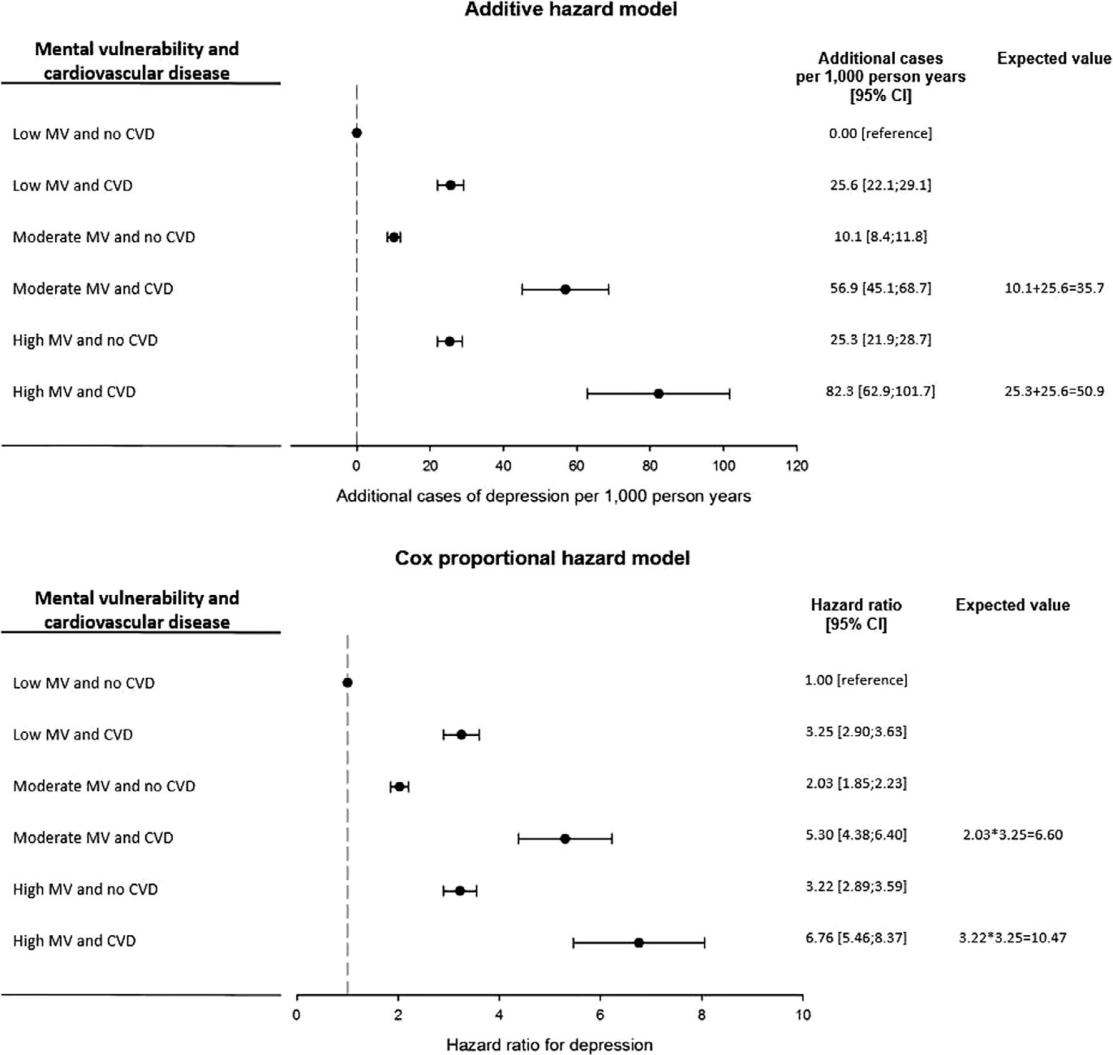
Adjusted associations between the combined variable of mental vulnerability (MV) and onset of cardiovascular disease for depression with one reference category of no mental vulnerability (MV) and no cardiovascular disease (CVD).

We explored whether mental vulnerability interacted with CVD for development of depression. Compared to individuals with no CVD and low mental vulnerability, both CVD and mental vulnerability separately and combined increased the risk estimates of developing depression (Figure 2). CVD combined with the highest level of mental vulnerability was associated with the highest risk estimate of 82.3 (95% CI: 62.9; 101.7) additional cases of depression per 1,000 person-years. Based on the individual effects of CVD (25.6 additional cases) and moderate mental vulnerability (10.1 additional cases), the expected joint effect on the additive scale is the sum of both exposures, thus, 35.7 additional cases per 1,000 person-years. However, the observed joint effect was 56.9 (95% CI: 45.1; 88.7) additional cases per 1,000 person-years. Similarly, the expected joint effect of CVD (25.6 additional cases) and high mental vulnerability (25.3 additional cases) would be 50.9 additional cases per 1,000 person-years, but the observed joint effect was 82.3 additional cases per 1,000 person-years. This expressed a synergistic interaction on the additive scale. Global tests of interactions were statistically significant (*p* value < 0.001). When we examined interaction on the multiplicative scale, the analysis yielded opposite results. The estimates from the Cox PH model showed antagonistic interactions on the multiplicative scale because the joint effects of CVD and higher levels of mental vulnerability were lower than the expected joint effects, that is products of the risk estimates. The antagonistic interaction was statistically significant (global test of interaction: *p* value < 0.001).

### Supplementary analyses

**Figure A3.**
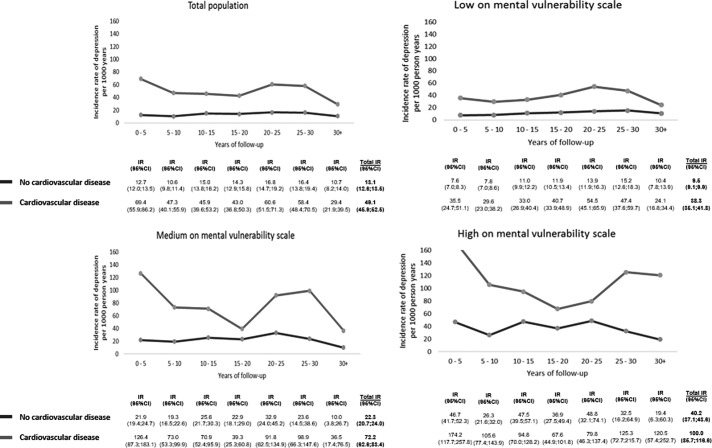
Crude incidence rates (IR) and 95% confidence intervals (95% CI) of depression in strata of individuals with low, moderate, and high mental vulnerabilities.

The rate in the reference population influences the estimations from the additive hazard model and Cox PH model differently because absolute differences are calculated on the additive scale, whereas ratios are calculated on the multiplicative scale. Figure A3 illustrates the rate of depression per 1,000 person-years of follow-up by CVD in the total population and in strata of individuals with low, moderate, and high mental vulnerability. The rate of depression was greater among individuals with CVD in all strata of mental vulnerability: low (CVD: 38.3, no CVD: 9.6), moderate (CVD: 72.2, no CVD: 22.3), and high (CVD: 100.0, no CVD: 40.2). Notably, the rate of depression among both individuals with and without CVD increased from individuals with low, through moderate to high mental vulnerability.

The findings were similar in the supplementary analyses in which depression was defined by prescription of antidepressants or hospital diagnosis, respectively (Table A2). Yet, the estimates of hospital-based diagnoses of depression from the additive hazard model were markedly lower than those for antidepressant medication due to a much lower incidence. Finally, when the study population was restricted to individuals without prior depression, the results were similar albeit with slightly less pronounced point estimates (Table A3).

## Discussion

In this cohort study of 19,856 Danish adults, there was a strong positive association between CVD and depression corresponding to 35.4 (95% CI: 31.7; 39.1) additional cases per 1,000 person-years. The relationship was only slightly attenuated after adjustment for confounders and mental vulnerability. We also found synergistic interaction between CVD and mental vulnerability in the additive hazard model where CVD seemed to cause 50.9 more cases of depression per 1,000 person-years than expected due to interaction between CVD and high mental vulnerability.

Our analyses confirm previous findings of a relationship between CVD and depression [2–5]. Mental vulnerability explained the same proportion of the relationship between CVD and depression as all other potential confounders combined including socio-demographic factors and health-related behaviors. The role of mental vulnerability may be explained by individuals who score higher on the mental vulnerability score are more susceptible to both conditions because they worry more and experience greater stress response when exposed to external stressors as previously suggested [9,10]. However, it is important to highlight that neither all the other potential confounder combined nor mental vulnerability explained much of the relationship between CVD and depression. Thus, the mechanisms linking CVD and depression still need to be explored further.

Test of interaction indicated that by higher levels of mental vulnerability, more CVD patients developed depression when analyzed on the additive scale. The greater than additive interaction indicates that targeting interventions toward mentally vulnerable patients with CVD, for example, providing more resources for rehabilitation programs to these patients, could lead to a larger reduction in number of patients who develop depression. This additive interaction between CVD and higher levels of mental vulnerability on the risk of depression may be explained by an increased stress response after CVD in individuals who are more mentally vulnerable. This explanation is in line with findings from a previous Danish study in which acute coronary syndrome patients who were exposed to additional stressors and lack of coping resources, reflected by specific patient characteristics, were more susceptible to develop depression after the event [3]. An American study has furthermore shown that exposure to adverse life events was associated with greater onset of depression among individual who scored high on neuroticism [30]; a personality trait which has also been shown to be partly related to mental vulnerability [7]. A previous Danish study highlights that a detected association between mental vulnerability and depression could be partly explained by the mechanisms linking neuroticism and depression [9].

The results from the two analytical models (additive hazard model and Cox PH model) conflict in the sense that the interaction results from the additive hazard model show that CVD patients, who are mentally vulnerable, are at an increased risk of developing depression on the additive scale. On the contrary, the interaction results from the Cox PH analysis show that it is protective for CVD patients to be mentally vulnerable in regard to subsequent depression risk on the multiplicative scale. The differences between the two analytical models can be explained by higher rates of depression among individuals without CVD (reference group) who have moderate and high mental vulnerability scores compared to the corresponding individuals with a low mental vulnerability score (Figure A3). As previously described, we believe that the results from the additive hazard model should be prioritized when we aim to identify whether more cases of depression can be treated or prevented by interventions focused on individuals who are exposed to both CVD and higher levels of mental vulnerability.

### Strengths and limitations

A major strength is the large study population of 19,856 randomly selected Danish men and women, which facilitated enough power to conduct interaction analyses. As recommended in psychiatric and epidemiological literature [25,27–29], interactions were investigated both on the additive and multiplicative scale in order to identify the greatest potential for intervention. This analytical approach was shown to be crucial in this study as the results differ between the additive and multiplicative models regarding identification of interactions. The analyses of CVD onset and depression onset ensured temporality between the exposure and the outcome. Access to extensive survey data linked with nation-wide register-based data enabled comprehensive adjustment of potential confounders including socio-demographic and behavioral factors. Information about CVD was obtained from the Danish National Patient Register that has a high quality with an acceptable coverage and validity except for unstable angina [31]. There is also a high agreement between register-based psychiatric diagnoses and research criteria for the depression diagnoses [32]. Furthermore, CVD and depression were assessed through registers independent of the participants´ ability to answer a questionnaire, which limits information bias. However, we cannot rule out the possibility that some degree of random misclassification of CVD and depression may have underestimated the association.

## Conclusion

Mental vulnerability explained a minor part of the relationship between CVD and depression risk. CVD caused more cases of depression among individuals who had a moderate or high mental vulnerability score than among individuals who had a low score. This suggests that healthcare professionals should be attentive to mentally vulnerable CVD patients who have the highest risk of developing depression.

## Data Availability

Researchers need to apply Statistics Denmark to access the anonymized dataset used in this study. Only aggregated data, where no identification of persons is possible, that is minimum five observations in each cell, can be removed from the server containing the data accessed through Statistics Denmark. Thus, we cannot provide an anonymized copy of the dataset as individuals may be identified based on the information in the data for example birthday, sex, depression diagnosis, and so on. Access to the data through Statistics Denmark is only granted for authorized research and analysis environments of a more permanent nature with a chief researcher and several researchers/analysts. Foreign researchers affiliated to a Danish authorized environment can also get access. Authorization is granted by the Director General. Please find more information in the document “Access to micro data at Statistics Denmark_2014” on https://www.dst.dk/en/TilSalg/Forskningsservice

## Appendix

**Table A1.** Mental vulnerability score based on baseline characteristics of the study population

	Mental vulnerability scale			
	Low	Moderate	High	Missing
All	15,238 [73.0]	2,966 [14.2]	1,652 [7.9]	1,014 [4.9]
Age				
Mean	48.1 [11.8]	46.9 [11.9]	46.6 [11.8]	49.3 [12.8]
Gender				
Females	7,333 [67.1]	1,883 [17.2]	1,128 [10.3]	586 [5.4]
Males	7,905 [79.5]	1,083 [10.9]	524 [5.3]	428 [4.3]
Education				
Short	3,229 [65.3]	824 [16.7]	598 [12.1]	293 [5.9]
Medium	8,179 [74.1]	1,549 [14.0]	799 [7.2]	516 [4.7]
Long	3,687 [79.7]	555 [12.0]	213 [4.6]	170 [3.7]
Missing	143 [55.4]	38 [14.7]	42 [16.3]	35 [13.6]
Marital status				
Married	10,809 [75.4]	1,904 [13.3]	943 [6.6]	671 [4.7]
Unmarried	2,492 [68.8]	556 [15.3]	388 [10.7]	188 [5.2]
Divorced	1,550 [65.7]	426 [18.1]	279 [11.8]	105 [4.5]
Widow/widower	387 [69.2]	80 [14.3]	42 [7.5]	50 [9.0]
Cohorts				
Monica I	2,963 [79.7]	499 [12.1]	281 [7.6]	24 [0.7]
Monica III	1,359 [70.6]	213 [11.1]	116 [6.0]	237 [12.3]
Inter99	4,348 [68.2]	1036 [16.3]	571 [9.0]	422 [6.6]
Health2006	2,052 [70.9]	446 [15.4]	243 [8.4]	153 [5.3]
DanFunD	4,516 [75.8]	822 [13.8]	441 [7.4]	178 [3.0]
Smoking				
Never	6,116 [76.4]	1,013 [12.7]	500 [6.2]	381 [4.8]
Former	4,271 [73.1]	832 [14.2]	432 [7.4]	310 [5.3]
Current	4,851 [69.1]	1,121 [16.0]	720 [10.3]	323 [4.6]
Alcohol consumption				
Mean [SD]	1.2 [1.5]	1.2 [1.6]	1.2 [2.0]	1.2 [1.4]
Physical activity				
Sedentary	2,527 [61.6]	762 [18.6]	592 [14.4]	220 [5.4]
Light	8,750 [73.4]	1,722 [14.4]	846 [7.1]	610 [5.1]
Moderate	3,718 [81.6]	457 [10.0]	205 [4.5]	175 [3.8]
Hard exercise	243 [85.0]	25 [8.7]	9 [3.2]	9 [3.2]
Body mass index				
Underweight [<21.5]	6,189 [75.5]	1,049 [12.8]	574 [7.0]	383 [4.7]
Normal weight [21.5–25.0]	2,232 [69.3]	506 [15.7]	330 [10.3]	151 [4.7]
Overweight [>25]	6,817 [72.1]	1,411 [14.9]	748 [7.9]	480 [5.1]
Systolic blood pressure				
First quintile	2,988 [68.9]	676 [15.6]	449 [10.4]	222 [5.1]
Second quintile	3,271 [72.6]	680 [15.1]	376 [8.3]	180 [4.0]
Third quintile	3,303 [74.5]	629 [14.2]	303 [6.8]	198 [4.5]
Fourth quintile	2,855 [74.7]	499 [13.1]	272 [7.1]	196 [5.1]
Fifth quintile	2,821 [74.8]	482 [12.8]	252 [6.7]	218 [5.8]
Pre diabetes				
No	15,061 [73.1]	2,927 [14.2]	1,622 [7.9]	994 [4.8]
Yes	177 [66.5]	39 [14.7]	30 [11.3]	20 [7.5]
Previous depression				
No	14,560 [74.8]	2,636 [13.5]	1,322 [6.8]	944 [4.9]
Yes	678 [48.2]	330 [23.4]	330 [23.4]	70 [5.0]

**Table A2.** Associations between onset of CVD and onset of depression

Cardiovascular disease	Rate of depression 95% CI	Additive hazard model	Cox proportional hazard model
		Adjusted^a^	Adjusted^a^
Onset of depression based on medicine			
All			
No	12.9 [12.5; 13.4]	0.00	1.00
Yes	48.8 [45.6; 52.2]	31.1 [27.4; 34.7]	2.78 [2.53; 3.06]
Mental vulnerability and onset of cardiovascular disease			
Low MV and no CVD	9.4 [8.9; 9.8]	0.00	1.00
Low MV and CVD	38.1 [34.9; 41.5]	25.3 [21.8; 28.8]	3.21 [2.87; 3.59]
Moderate MV and no CVD	22.0 [20.4; 23.7]	10.0 [8.3; 11.7]	2.04 [1.86; 2.24]
Moderate MV and CVD	71.8 [62.1; 82.8]	56.5 [44.6; 68.4]	5.24 [4.33; 6.34]
High MV and no CVD	39.7 [36.6; 43.1]	25.0 [21.7; 28.4]	3.25 [2.92; 3.63]
High MV and CVD	98.5 [84.5; 114.9]	79.2 [60.2; 98.2]	6.55 [5.28; 8.12]
Onset of depression/anxiety based on hospitalization			
All			
No	1.3 [1.1; 1.4]	0.00	1.00
Yes	17.1 [15.5; 18.8]	3.5 [3.1; 3.9]	2.56 [1.98; 3.32]
Mental vulnerability and onset of cardiovascular disease			
Low MV and no CVD	0.9 [0.7; 1.0]	0.00	1.00
Low MV and CVD	12.4 [10.8; 14.1]	2.2 [1.2; 3.2]	3.23 [2.28; 4.57]
Moderate MV and no CVD	2.0 [1.6; 2.5]	0.8 [0.3; 1.3]	2.02 [1.51; 2.70]
Moderate MV and CVD	22.8 [18.6; 27.9]	3.2 [0.8; 5.6]	3.87 [2.22; 6.74]
High MV and no CVD	4.1 [3.3; 5.1]	2.5 [1.6; 3.5]	3.51 [2.60; 4.74]
High MV and CVD	35.6 [29.6; 42.8]	9.0 [4.6; 13.5]	7.56 [4.73; 12.07]

Abbreviations: CVD, cardiovascular disease; MV, mental vulnerability.

a Adjusted for age, sex, educational level, marital status, smoking status, alcohol consumption, physical activity, body mass index, systolic blood pressure, diabetes, previous depression, study cohorts, and mental vulnerability.

**Table A3.** Associations between onset of CVD and onset of depression excluding individuals with prior depression

Cardiovascular disease	Additive hazard model	Cox proportional hazard model
	Adjusted^a^	Adjusted^a^
Onset of depression for individuals without prior depression		
All		
No	0.00	1.00
Yes	27.0 [23.6; 30.3]	2.64 [2.40; 2.91]
Mental vulnerability and onset of cardiovascular disease		
Low MV and no CVD	0.00	1.00
Low MV and CVD	24.4 [21.1; 27.8]	3.21 [2.87; 3.59]
Moderate MV and no CVD	10.3 [8.6; 11.9]	2.10 [1.91; 2.32]
Moderate MV and CVD	45.9 [44.3; 47.6]	4.81 [3.95; 5.86]
High MV and no CVD	24.6 [21.4; 27.8]	3.56 [3.18; 3.98]
High MV and CVD	59.6 [43.6; 75.6]	5.72 [4.53; 7.22]

Abbreviations: CVD, cardiovascular disease; MV, mental vulnerability.

a Adjusted for age, sex, educational level, marital status, smoking status, alcohol consumption, physical activity, body mass index, systolic blood pressure, diabetes, previous depression, study cohorts, and mental vulnerability.
